# The Chemical Transformation of the Cellular Toxin INT (2-(4-Iodophenyl)-3-(4-Nitrophenyl)-5-(Phenyl) Tetrazolium Chloride) as an Indicator of Prior Respiratory Activity in Aquatic Bacteria

**DOI:** 10.3390/ijms20030782

**Published:** 2019-02-12

**Authors:** Josué Villegas-Mendoza, Ramón Cajal-Medrano, Helmut Maske

**Affiliations:** 1 Facultad de Ciencias Marinas. UABC, Carretera Tijuana-Ensenada km 106, Ensenada, Baja California, CP 22860, Mexico; 2 CICESE, Carretera Tijuana-Ensenada No. 3918, Ensenada, Baja California, CP 22860, Mexico; rcajal@uabc.edu.mx (R.C.-M.); hmaske@cicese.mx (H.M.)

**Keywords:** prokaryotes, respiration, INT toxicity

## Abstract

In the ocean, the prokaryote respiration rates dominate the oxidation of organics, but the measurements may be biased due to pre-incubation size filtration and long incubation times. To overcome these difficulties, proxies for microbial respiration rates have been proposed, such as the in vitro and in vivo estimation of electron transport system rates (ETS) based on the reduction of tetrazolium salts. INT (2-(4-Iodophenyl)-3-(4-Nitrophenyl)-5-(Phenyl) Tetrazolium Chloride) is the most commonly applied tetrazolium salt, although it is toxic on time scales of less than 1 h for prokaryotes. This toxicity invalidates the interpretation of the rate of in vivo INT reduction to formazan as a proxy for oxygen consumption rates. We found that with aquatic bacteria, the amount of reduced INT (F; µmol/L formazan) showed excellent relation with the respiration rates prior to INT addition (R; O_2_ µmol/L/hr), using samples of natural marine microbial communities and cultures of bacteria (*V. harveyi*) in batch and continuous cultures. We are here relating a physiological rate with the reductive potential of the poisoned cell with units of concentration. The respiration rate in cultures is well related to the cellular potential of microbial cells to reduce INT, despite the state of intoxication.

## 1. Introduction

Respiration is the main energy acquisition mechanism by which all living creatures transform organic matter to CO_2_. Planktonic organisms in the pelagic ocean respire a large portion of all the organic matter produced; the heterotrophic bacterioplankton alone contribute about 40% to all plankton respiration in the ocean [1]. The measurement of bacterial respiration is still problematic, and it is currently considered a significant obstacle to produce accurate budgets for the carbon cycle in the ocean [1,2]. The two most common techniques used to measure plankton respiration in the ocean, are the oxygen consumption in the dark by the whole or fractionated plankton communities, or the measurement of the electron transport system (ETS) either in vitro (ETS*_vitro_*) [3]; or in vivo (ETS*_vivo_*) [4]. To measure the prokaryote respiration monitoring oxygen consumption in the dark, the prokaryotes in the sample have to be separated before the incubation, typically through size filtration. It has been reported that pre-incubation size filtration changes the predatory pressure [1], releases organic compounds [5] and can induce changes in community structure [6,7]. These alterations lead to changes of bacterial respiration in aquatic samples, particularly in marine samples that typically need long incubation periods. Aranguren-Gassiz et al. (2012) [8] argued that these limitations lead to the overestimation of microbial respiration rates. The ETS*_vitro_* method [3] allows us to evaluate the potential plankton respiration with high sensitivity [9], but suffers the drawback that when it is applied to prokaryotes, the ratio of ETS*_vitro_* to oxygen respiration depends on the physiological state of the prokaryotes [10,11]. ETS has also been measured in vivo in microbial oceanography [4]. ETS*_vivo_* involves incubating marine plankton with INT without the addition of enzymatic substrates, arguing that the reactions are occurring at natural substrate levels. The sensitivity of ETS*_vitro_* was reported to approach the ETS*_vitro_* method. It has recently been found that the toxic effect of INT would not allow for the estimation of the plankton respiration rate with the ETS*_vivo_* method [12]. The toxicity in marine prokaryote cultures led to a decrease in respiration rates with an increase in INT concentrations (0.05 to 1 mmol/L), showing that the initial rapid INT reduction rate to formazan rapidly decreased and terminated after about 1 h [12]. For eukaryotes, the oxygen consumption by respiration and the INT reduction to formazan is also decreased, but this decrease occurs over a longer period of time than for prokaryotes [12]. In prokaryotes, the respiratory electron transport system is located in the cell membrane [13,14] in Gram negative [15] and Gram positive bacteria [16] facilitating the INT reduction. We always find the toxic effect of INT on microbial respiration, as shown below in one previously unpublished example. In eukaryotes, the extracellular INT has to diffuse to the mitochondria to be reduced by the ETS. This restricts the potential reduction and the toxic effect of INT, complicating the interpretation of in vivo INT reduction. Martínez-García et al. (2009) [4] showed that in eukaryotes, the formazan production is only stopped after several hours, indicating a delayed toxic effect. The ETS*_vivo_* method as it has been applied up to this day as a rate measurement [4,17,18,19] presents several potential problems: INT is toxic because it interferes with the respiration chain in prokaryotes and eukaryotes, the kinetics of poisoning for prokaryotes and eukaryotes have very different time scales, but in mixed natural populations they occur simultaneously in the same sample, and apart from the respiratory chain other cell components might also reduce INT [20,21].

Given the limitation of the ETS*_vivo_* method, we tried a different approach in marine prokaryote samples, where we empirically relate the total amount of INT reduced into formazan (F, µmol/L) to the oxygen respiration rate prior to the INT addition. We propose that during short term incubations (1 h), the ETS activity and reducing metabolites present in the cell membrane of prokaryotes are reducing the INT to formazan crystals until this reduction potential is exhausted. The amount of INT reduction during this short period is proportional to the pre-incubation ETS activity that set the rate of oxygen respiration. In other terms, the amount of the reduced poison allows us to estimate a physiological rate before poisoning.

In this study, we measured the short term INT-formazan production and oxygen respiration in marine bacteria cultures within a wide range of growth rates and different temperatures. The results of the formazan production during short term incubations yielded a statistical significant relationship that can be used as a proxy for bacterial respiration in aquatic environments.

## 2. Results

### 2.1. The Respiration (R) to In Vivo Formazan Production (F) Relationship.

**Figure 1:**
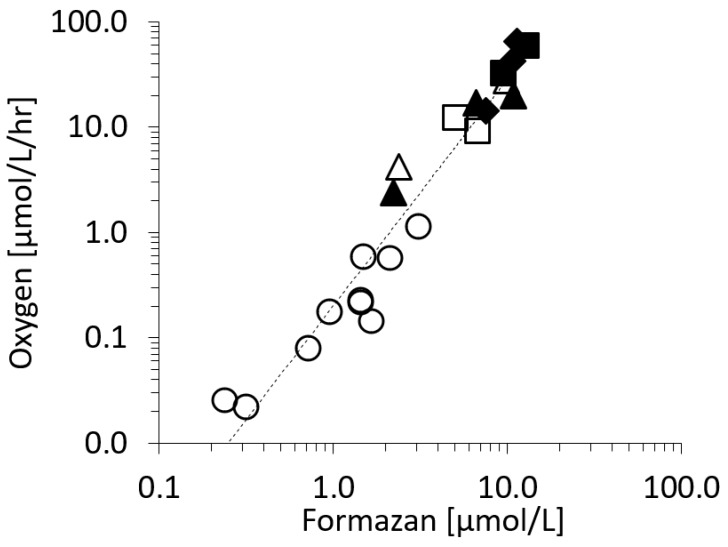
Respiration rate versus formazan production of communities of marine prokaryotes in continuous cultures, and *V. harveyi* in batch cultures, △ 10 °C, □ 15 °C, ○ 18 °C, ▲ 20 °C, ■ 25 °C, ◆ 28 °C.

*R* (µmol O_2_/L/hr) and *F* (µmol formazan/L) were measured using continuous cultures of marine bacteria assemblages and batch cultures of *V. harveyi.* They showed a clear pattern of exponential increase with increasing specific growth rate and temperature (Figure 1). *R* versus *F* was compared in Figure 1, resulting in:

Continuous line; *R* = 0.20 *F*^2.15^ r^2^ = 0.93; *p* < 0.05 (1)

**Figure 2:**
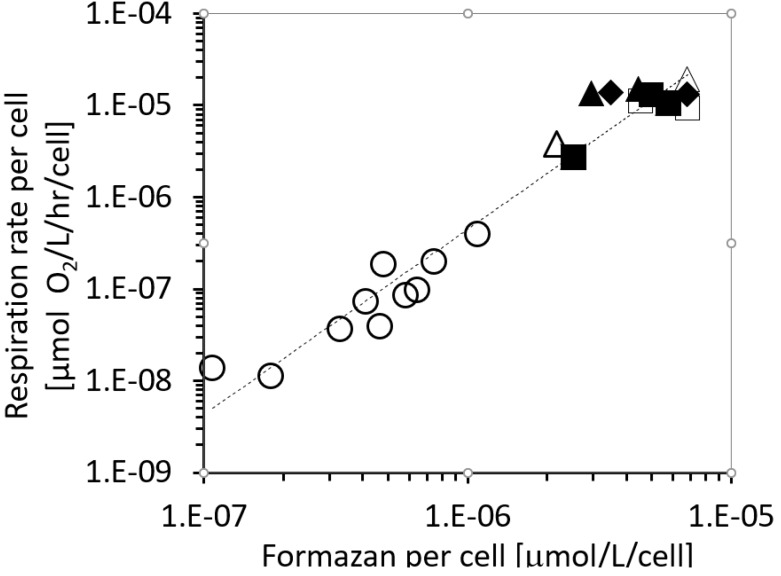
Respiration rate per cell versus formazan per cell of marine prokaryotes in continuous cultures, and *V. harveyi* in batch cultures, discontinuous line *y* = 0.39*x*1.9, r^2^ = 0.96, *p* ≤ 0.05. △ 10 °C, □ 15 °C, ○ 18 °C, ▲ 20 °C, ■ 25 °C, ◆ 28 °C.

Thereafter, the abundance normalized rates of oxygen respiration and formazan formation were calculated (Figure 2):

The data showed a similar general pattern, but for the *V.harveyi* data less change of oxygen respiration rate in relation to formazan formation than expected was found. The data can be found in the Supplementary Table S1.

### 2.2. Oxygen Consumption and Formazan Production Relationship with Specific Growth Rate and Temperature

**Figure 3:**
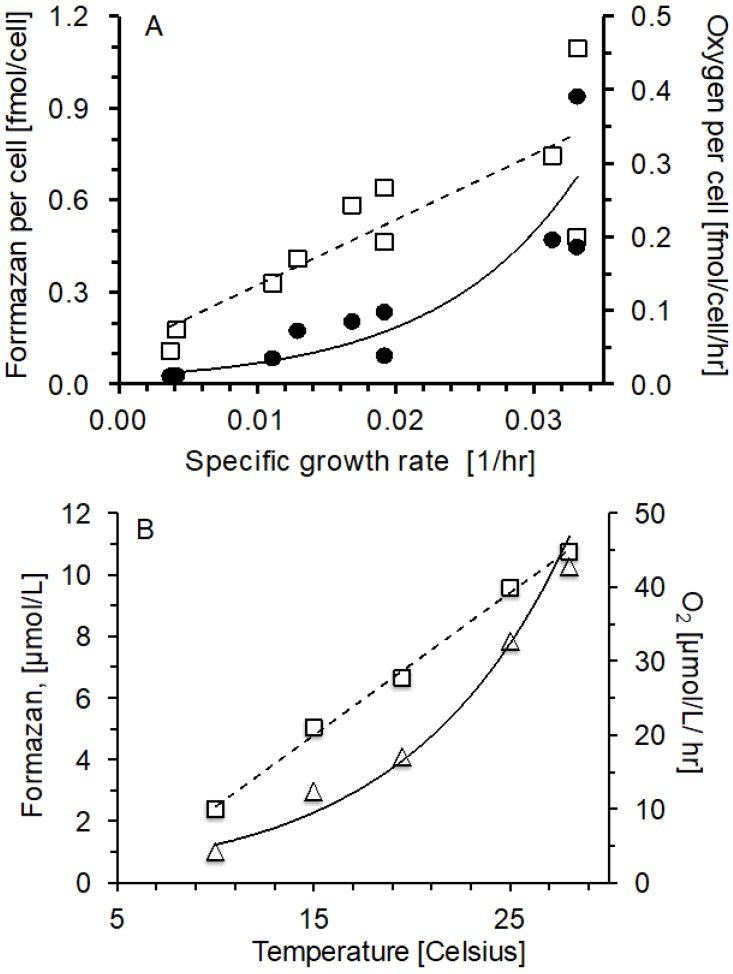
Oxygen consumption and formazan vs. specific growth rate and temperature. (**A**) Assemblages of marine prokaryotes in continuous cultures, formazan production per cell (□, dashed line, *y* = 21.4*x* + 0.11, r^2^ = 0.69, *p* ≤ 0.05), oxygen consumption per cell (● continuous line, *y* = 0.01*e*^98.7*x*^, r^2^ = 0.87, *p* ≤ 0.05). (**B**) *V. harveyi* in batch cultures, oxygen consumption (△, continuous line, *y* = 1.51*e*^0.12*x*^, r^2^ = 0.97, *p* ≤ 0.05) and formazan production (□, dashed line, *y* = 0.46*x* − 2.16, r^2^ = 0.99, *p* ≤ 0.05). Error bars are not shown because they are too small.

Marine bacterial communities growing in organic substrate limited continuous cultures at specific growth rate from 0.004 to 0.034 1/hr and in *V. harveyi* batch cultures from 1.57 to 7.49 1/hr showed a significant positive relationship between specific growth rate and oxygen consumption rate per cell (r^2^ = 0.87 *p* ≤ 0.05) and formazan per cell production (r^2^ = 0.69, *p* ≤ 0.05) (Figure 3A). Also for the batch cultures of *V. harveyi* growing at different temperatures (from 10 to 28 °C) the formazan production increased with higher temperatures and specific growth rates at variance with an oxygen consumption rate that increased exponentially with temperature (Figure 3B):

Figure 3A,B show marine prokaryotes populations growing under different degrees of nutrient limitation at 18 °C, and the marine bacterium *V. harveyi* during the exponential growth phase in batch cultures within a temperature range from 10 to 28 °C (Supplementary Table S1). The different temperatures led to a range of physiological adaptations where R could be compared with F using 1 h incubations with 0.5 mmol INT/L.

### 2.3. Formazan Production and the Rate of Formazan Production in V. Harveyi Batch Cultures.

**Figure 4:**
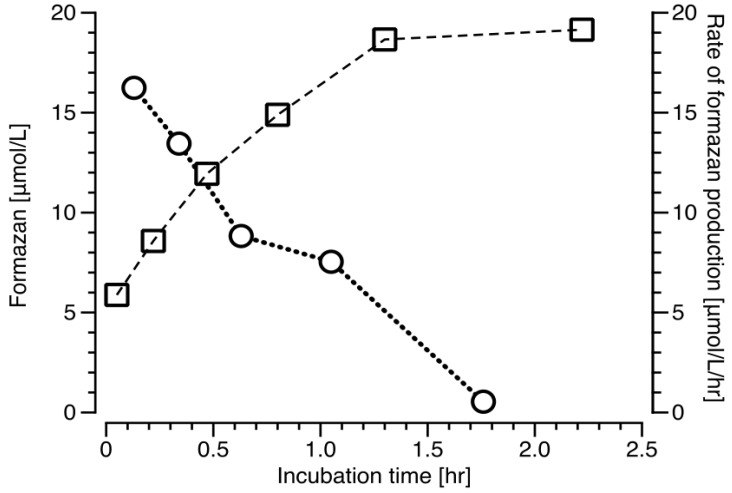
Formazan in *V. harveyi* batch cultures amended with 0.5 mM INT (□), and rate of formazan production (○).

The *V. harveyi* batch cultures amended with INT show a steady increase in the formazan production rate, achieving the maximum at 1.3 h of incubation. In contrast, the formazan production decreases rapidly within the first 30 min approaching zero at 1.76 h of incubation (Figure 4).

**Figure 5:**
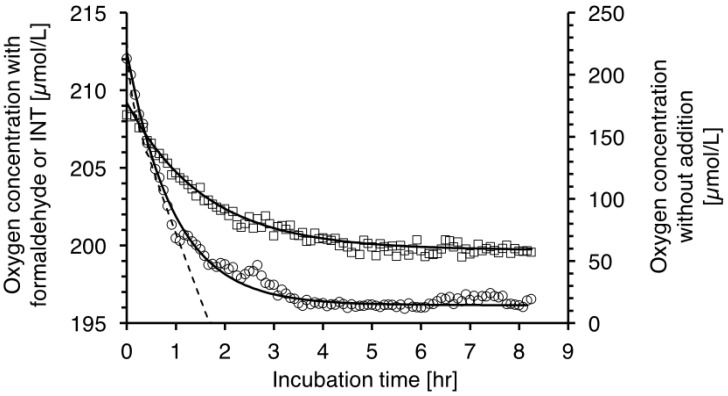
Oxygen concentration over time in enclosed *V. harveyi* cultures. Note difference in ordinate scales: Left ordinate: Oxygen concentration after addition of INT (□) and formaldehyde (○). Continuous lines represent the exponential decay function fitted to the data including the baseline calculated from the concentrations at > 6 h. Right ordinate, corresponding to the dashed line to avoid cluttering the figure: The oxygen concentration without additions to the culture.

The time scale of formazan formation corresponds approximately with the kinetic constant calculated for the oxygen consumption in *V. harveyi* cultures with INT added (Figure 5).

## 3. Discussion

### 3.1. Toxicity of INT

The toxicity of tetrazolium salts has been reported for both eukaryotic and prokaryotic Gram positive and Gram negative cells. For instance for prokaryotic cells, May et al. (1960) [22] demonstrated that growth of some bacteria (*E. coli*, *Sal*. *typhimurium, P. vulgaris* and *Sh. sonnei*) is inhibited by tetrazolium salts, and more effectively by ditetrazolium salts than monotetrazolium salts like INT. As discussed in (Villegas-Mendoza [12] and literature therein), bacterial production, growth and glucose uptake is inhibited by tetrazolium salts. The interference of INT with cellular respiration could be through different mechanisms like substrate competition with oxygen, permanent blocking of enzyme acceptor sites or lack of cellular energy equivalents. In Figure 1, we show the direct effect of INT on respiration rate for the marine bacteria *V. harveyi*. The time when oxygen concentration approached 1/e of the initial concentration was 1.57 h for INT and 0.95 h for formaldehyde treated *V. harveyi* cultures. The mechanisms of reduction of the tetrazolium salts like INT and CTC by *E. coli* have been studied by Smith and McFeters (1997) [21]. The authors demonstrated that the reduction of the tetrazolium salts INT and CTC by aerobic dehydrogenases prior to ubiquinone in the respiratory chain of *E. coli*. INT, was also reduced also by ubiquinone and conceivably by cytochromes b_555_, b_556_ within the ETS. Using *E. coli* anaerobic cultures, they also found significant reduction of INT. Our epifluorescence and transmission light microscope observations (not shown) and previous observations [12] confirmed the position of the formazan crystals at the cell wall where the ETS system is placed; see also [23]. Because the INT reduction by prokaryotic cells takes place close to the ETS location, we would like to argue that the formazan production gradually depletes the electron sources within the ETS.

### 3.2. The ETS In Vitro and In Vivo Methods to Evaluate Respiration Rate

The INT salt reduction rate in vitro has a long history to use as a respiration rate proxy [3,9,10,24,25]. This in vitro method (ETS*_vitro_*) used to estimate the oxygen consumption of plankton is attractive due to its high sensitivity, and therefore might help to overcome the limitations of monitoring oxygen consumption. But only few publications have applied ETS*_vitro_* to marine prokaryotes [10,11]. These studies found a variable ETS*_vitro_* activity to oxygen respiration rate ratio depending on the physiological state of the prokaryotes. For instance, they found variation in R/ETS among five different bacterial species, but a relatively constant R/ETS among the same species if senescent or growing populations were considered separately. The R/ETS ratio is used to indicate electron flow per mole of formazan produced (mol O_2_ equivalent vs mol formazan equivalent) in the cells.

The in vivo tetrazolium reduction rate method has been used for several decades in other fields of microbiology, e.g., soil microbiology [17]. Martínez-García et al. 2009 and Martínez-García et al. 2013 [4,26] proposed that the in vivo ETS evaluation method using the tetrazolium salt INT in which no substrate is added to the sample and the formazan produced by cell levels of NADH, is collected on a filter after incubation. Formazan production rate (µmol/L/hr) is estimated from the incubation time, which would increase experimentally as the metabolic activity decreases. This apparently simple rate method [4] has been used in an oceanic basin scale comparison of microbial allometry [27]. But considering that the timescales of poisoning by INT are different for prokaryotes and eukaryotes with consequences in the interpretation of formazan production rates, and that the differences in timescales will most likely then be related to cell size, we conclude that the rate method can easily lead to misinterpretations.

### 3.3. The INT Reduction Potential Method

We suggest that the method developed here is based on a different principle; namely on the total amount of formazan produced on the membranes of the bacterial cells (µmol oxygen/L/hr vs µmol formazan/L) and empirically related to the oxygen consumption rate of bacterial cultures. We have previously shown that INT is toxic to prokaryotes and that the respiration rate decreases with incubation time [12]. Moreover, the oxygen consumption rates decrease on similar time scales with either formaldehyde or INT additions (Figure 1). Because the respiration rates rapidly decrease with time in all samples, it would be difficult to define a representative rate of INT reduction, which has been the approach in previous publication using INT reduction in vivo [4,8]. Also, regarding the proposed method, there is no conceptual basis for an interpretation of the stoichiometric relationship between reduced INT and the rate of oxygen consumption (R/ETS) as applied in the ETS*_vitro_* method [10,11]. Our method is based on the concept that the formazan produced (*F*) represents the potential cell capacity to reduce INT in the prokaryote cells membrane, and is responsible for reducing the INT on time scales of 1 h. We propose that the potential cell capacity to reduce INT can be used as a proxy for the respiration rate (Equation (1)). Figure 4 shows that in a bacterial culture 85% of the total formazan is produced within 1 h. Although in continuous cultures with natural bacteria inoculum the rate of change was sometimes slower, still after 1 h the great majority of the total formazan had been produced. We explicitly suggest to use this method only for the estimation of prokaryotes, because the internal cell organization of eukaryotes complicates the interpretation of INT reduction results.

When all our data of *R* vs. *F* (µmol oxygen/L/hr vs µmol formazan/L) are graphed (Figure 2) a consistent trend is found (Equation (1) and Figure S1):

The formazan concentration produced (*F*, µmol/L) during a short period (1 h) is related to the oxygen respiration rate before the exposure of the cells to INT. The per cell respiration rate plotted on Figure 2, shows a similar pattern as Figure 1, suggesting that the obtained relationship is not forced by cell abundance.

The *R* to *F* relationship holds for marine bacterial assemblages growing at different growth rates in continuous cultures (Figure 3A) and for *V. harveyi* batch cultures growing at different growth rates and temperatures (Figure 3B). *R* and *F* increased with growth rate and temperatures as expected. In both types of cultures, the oxygen consumption rate increased in proportion more than the formazan formation. In fact, the kinetics of *F* shows a linear increase with specific growth rate and temperature (Figure 3A,B) different from the exponential pattern for *R* (Figure 3B). However, we believe that the linear relationship for *F* in the bacterial cultures receiving the INT additions is probably related to the initial INT reduction capacity of the bacterial cell at the onset of the experiment, which would be related to the different ETS size of the bacteria at different growing rates and physiological conditions. The chemostat experiments (Figure 3A) covered limiting growth conditions, the range of growth rates (0.004 to 0.033 1/hr, Figure 3A) were similar to oceanic rates. Our bacterial continuous cultures are not monospecific cultures, but included different marine bacteria communities with different diversities. In both batch and continuous cultures, the cell densities were similar and close to oceanic concentrations (Supplementary Table S1). Our data covers a wide range of respiration rate, the batch cultures of the marine bacteria *V. harveyi* showed much higher respiration rates than the marine prokaryotes assemblages growing in substrate limited chemostats, demonstrating that our method is applicable to very different sample types.

Above we mentioned problems associated with pre-incubation size filtration in the measurement of the respiration of microbes in the ocean using oxygen consumption. Ward (1984) [5] had already demonstrated how impacting pre-incubation size filtration can be to the plankton physiology during incubation and Aranguren-Gassis et al. (2012) and Martínez-García et al. (2013) [8,26] had also demonstrated an overestimation of oxygen consumption for pre-incubated filter size fractionation. Our proposed protocol allows us to separate prokaryotes and eukaryotes in the samples after incubating for 1 h with INT without perturbing the state of the sample during incubation. A potential source of error of our method might be the loss of formazan crystals when the sample is filtered through 0.2-µm filter at the end of the incubation, but Villegas-Mendoza et al. (2015) [12] found that after incubation <4% of the formazan went through the 0.2 µm polycarbonate filter.

In summary, we have developed a protocol to estimate prokaryote respiration rate based on the total amount of formazan produced (*F* µmol/L) from the INT reduction at the surface of prokaryote cells after 1 h of incubation. *F* is empirically related to *R* (µmol O_2_/L) resulting in a useful relationship that would allow the use of *F* as a proxy for respiration rate in aquatic environments during short incubations time reducing potential artifacts from prolonged incubations. The method is attractive because it avoids long incubation times reducing potential artifacts from prolonged incubations.

## 4. Materials and Methods

### 4.1. Sample Collection

The marine bacteria inoculum was obtained fresh for each batch culture and continuous cultures experiments by collecting 250 mL of surface coastal seawater in polycarbonate bottles. Sample location was Bahía Todos Santos (31° 51 N, 116° 40 W) between June 2011 and July 2014. The samples were gently filtered (<34.5 kpa) through 0.8 polycarbonate filters (Poretics Corporation, Livermore, CA, USA) and the filtrate was used as inoculum.

### 4.2. Continuous Culture Preparation

The continuous cultures were prepared following Cajal-Medrano and Maske (2005) [28]. 20 L of culture media was prepared each time using aged seawater, filtered through GF/F filters, bubbled with an ozone stream for 24 h at 160 mL/min. The amount of ozone used was not quantified, but the gas leaving the culture medium had a very strong smell of ozone. Afterward 0.8 g/L of activated charcoal (cat. 05105) from Sigma-Aldrich (St. Louis., MO, USA) was added for 24 h and removed subsequently by filtration using 47 mm GF/F filters at 101.3 kPa. Inorganic nutrients and glucose as organic carbon source were then added (NH_4_Cl 30 µmol/L, KH_3_PO_4_ 5 µmol/L, FeCl_3_ 0.4 µmol/L and 20 µmol/L of glucose). Subsequently, the culture media was acidified by bubbling with CO_2_, autoclaved for 1 h at 15 psi, cooled down to room temperature and bubbled with sterile air to replenish O_2_ back to saturation. All containers and tubing used for the chemostat apparatus were teflon or silicon [28]. The continuous cultures were aseptically inoculated in sterile 2-L chemostat-growing chamber. The inoculated culture was left for 24 h, and then diluted at different specific dilution rates (0.004 to 0.033 1/hr). All cultures were stirred and run in the dark at the same temperature (18 °C). Sample collection for bacterial abundance, O_2_ concentration and formazan production in the chemostats was done when a steady state was reached. A steady state was defined by a constant bacterial abundance varying approximately 20% in cell abundance.

### 4.3. Batch Cultures of V. harveyi

The marine bacterium *V. harveyi* (from Dr. D. Bartlett, Scripps Institute of Oceanography, San Diego, CA, USA) growing in ZoBell liquid media was used to measure formazan production and oxygen consumption.

### 4.4. Bacterial Abundance

20 mL samples from chemostats or batch cultures were fixed with buffered formaldehyde (2% final solution) and a 0.2 to 0.5 mL sample volume was incubated with DAPI and filtered immediately on 0.2 µm black polycarbonate filters (Poretics). For each sample, a total of 10 fields were counted for a total of >300 cells [29] using an epifluorescence microscope (Carl Zeiss, Jena, Germany) equipped with a X100 objective and a 175W xenon lamp (Lambda LS, Sutter, Novato, CA, USA) connected through a liquid light guide.

### 4.5. Oxygen Consumption Determination

Because small water volumes from continuous cultures are needed, the Winkler spectrophotometric method as described by Roland et al. (1999) [30] was used for oxygen measurements taken from the culture vessel and the growing media. 20 mL samples were collected from the culture and medium vessels through Teflon tubing into scintillation vials with conical shaped plastic liners (Catalog No. 03-337-7 Fisher), allowing to close the lids without oxygen introduction, and minimizing the sample volume for spectrophotometric Winkler analysis. Immediately after overflowing three times the sample volume, the samples were fixed adding 1 mL of Winkler A and B solutions, prepared following JGOFS protocol (1996) [31]. When applied to 20 mL samples in scintillation vials, the Winkler spectrophotometric method has a coefficient of variation of 0.87 to 2.7%. The rate of oxygen consumption was calculated at culture steady state from the difference between the oxygen concentration in the media, and in the culture and multiplied by the dilution rate. The Winkler spectrophotometric method was also applied to batch cultures samples growing at high rates. For the INT toxicity experiments, the oxygen consumption rate was measured using Planar Oxygen-Sensitive Spots, SP-PSt3-NAU-YOP along with the Fibox 4 system (PreSens, Regensburg, Germany) or sensitive optode [32,33] using an oxygen-dipping probe (DP-PSt3-YOP).

### 4.6. The In Vivo Formazan Formation Measurement

The ETS in vivo method was used as described in Villegas-Mendoza et al. (2015) [12]. Briefly, samples from continuous and batch cultures of 20 to 100 mL were incubated with INT (0.5 mmol/L final concentration) with incubation periods of less than 1 h. Samples were then filtered through 0.2 µm polycarbonate filters to collect the cells and the formazan crystals. These filters were immediately preserved (-20 Celsius, <2 days) or immediately extracted with 1.5 mL propanol with a homogenizer (Beadbeater, Cole-Parmer, Vernon Hills, IL, USA, 600 s at 5000 rpm). Blanks were prepared by killing samples with a 2% final solution of formaldehyde about 1 h before INT addition. Triplicate blanks and samples were run at constant temperature range from 10 to 28 °C for bacterial batch cultures and 18 °C with bacterial continuous cultures. The blanks were subtracted from the sample value. Formazan concentration was measured at 485 nm in a double beam spectrophotometer (Perkin Elmer Lambda 40, Waltham, MA, USA). Formazan concentration was quantified based on calibration curves that were prepared using 1-(4-Iodophenyl)-5-(4-nitrophenyl)-3-phenylformazan from Sigma-Aldrich (cat. 17375).

## 5. Conclusions

We are here relating a physiological rate with the reductive potential of the poisoned cell. When we tried to use this method to estimate the respiration rates in natural samples, we found that the reduced INT was greater than expected. We conclude that in principle the cellular potential to reduce INT is well related to the current respiration rate.

## Supplementary Materials

Supplementary materials can be found at http://www.mdpi.com/1422-0067/20/3/782/s1.

## Abbreviations

F	Formazan; μmol/L
R	Respiration rates; μmol/L/hr
ETS	Electron transport system
INT	2-(4-Iodophenyl)-3-(4-Nitrophenyl)-5-(Phenyl) Tetrazolium Chloride
