# Supplementary material for: The Chemical Transformation of the Cellular Toxin INT (2-(4-Iodophenyl)-3-(4-Nitrophenyl)-5-(Phenyl) Tetrazolium Chloride) as an Indicator of Prior Respiratory Activity in Aquatic Bacteria
Source: Int J Mol Sci. 2019 Feb 12;20(3):782. doi: 10.3390/ijms20030782 (PMC6387158; doi:10.3390/ijms20030782)
Supplement: Supplementary file 1 [file ijms-20-00782-s001.pdf]

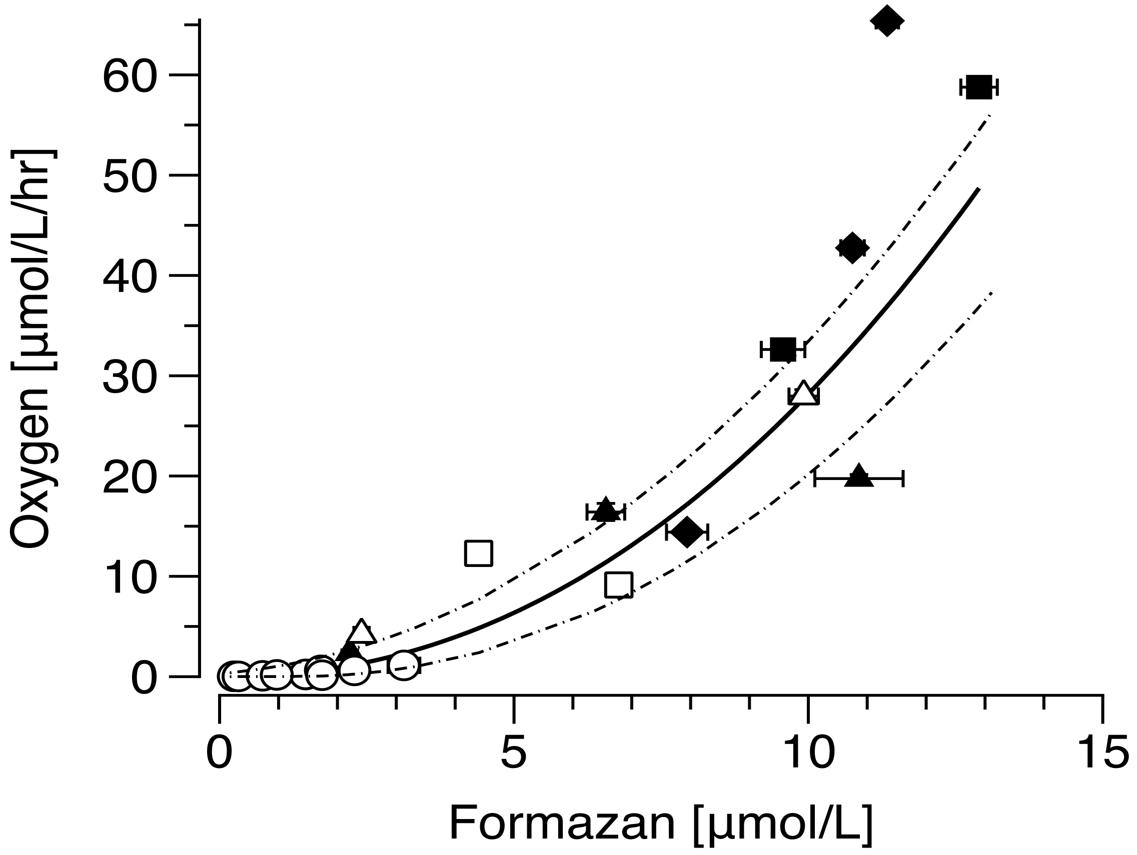

Supplementary Fig. S1. Respiration rate versus formazan production of communities of marine prokaryotes in continues cultures and *V. harveyi* in batch cultures. These are the same data as in Fig. 1 and 2 but on linear scale. Continuous line; $R = 0.20\ F^{2.15}$, $r^2 = 0.93$, confidence interval ($p \leq 0.05$) indicated by broken lines, symbols for temperature △10°C, □15°C, ○18°C, ▲20°C, ■ 25°C, ◆ 28°C.

Villegas-Mendoza et al. 2019.

Supplementary Table S1. Oxygen consumption and formazan production in batch and continues cultures at different temperature.

Sample	Temperature °C	Specific growth rate 1 / hr	Cell concentration cell / mL	O_2 respiration µmol / L / hr	Formazan µmol / L
V. harveyi				20.04	10.34
	20	0.4	1.88E+06	19.47	11.39
V. harveyi				9.19	6.82
	15	0.1	9.92E+05	9.10	6.74
V. harveyi				27.51	9.74
	10	0.7	1.46E+06	28.43	10.09
V. harveyi				14.49	8.19
	28	0.6	1.10E+06	14.33	7.69
V. harveyi				2.58	2.17
	20	0.5	8.76E+05	2.17	2.25
V. harveyi				32.97	9.83
	25	4.7	2.16E+06	32.27	9.31
V. harveyi				15.82	6.33
	19.5	3.0	1.33E+06	17.02	6.79
V. harveyi				12.43	4.41
	15	2.1	1.11E+06	12.14	4.4
V. harveyi				4.27	2.5
	10	1.6	1.10E+06	4.14	2.32
Bacterial Communities				0.03	0.17
	18	0.004	2.22E+06	0.02	0.29
					0.26
Bacterial Communities				0.02	0.23
	18	0.004	1.78E+06	0.02	0.29
					0.43
Bacterial Communities				0.23	1.63
	18	0.17	2.50E+06	0.2	1.48
				0.21	1.27
Bacterial Communities				0.23	1.29
	18	0.019	2.27E+06	0.21	1.43
				0.23	1.65
Bacterial Communities				1.13	3.34
	18	0.033	2.86E+06	1.1	3.24
				1.13	2.83
Bacterial Communities				0.07	0.8
	18	0.011	2.21E+06	0.08	0.71
				0.08	0.68
Bacterial Communities				0.18	0.82
	18	0.013	2.36E+06	0.16	1.1
				0.17	0.98
Bacterial Communities				0.66	2.24
	18	0.031	2.88E+06	0.57	2.34
Bacterial Communities				0.62	1.75
	18	0.033	3.13E+06	0.64	1.69
Bacterial Communities				0.17	1.74
	18	0.019	3.62E+06	0.12	1.74
